# Genome-wide association study to identify candidate loci and genes for Mn toxicity tolerance in rice

**DOI:** 10.1371/journal.pone.0192116

**Published:** 2018-02-09

**Authors:** Asis Shrestha, Ambrose Kwaku Dziwornu, Yoshiaki Ueda, Lin-Bo Wu, Boby Mathew, Michael Frei

**Affiliations:** 1 Institute of Crop Science and Resource Conservation (INRES) Plant Nutrition, University of Bonn, Bonn, Germany; 2 Institute of Crop Science and Resource Conservation (INRES) Plant Breeding, University of Bonn, Bonn, Germany; Aberystwyth University, UNITED KINGDOM

## Abstract

Manganese (Mn) is an essential micro-nutrient for plants, but flooded rice fields can accumulate high levels of Mn^2+^ leading to Mn toxicity. Here, we present a genome-wide association study (GWAS) to identify candidate loci conferring Mn toxicity tolerance in rice (*Oryza sativa* L.). A diversity panel of 288 genotypes was grown in hydroponic solutions in a greenhouse under optimal and toxic Mn concentrations. We applied a Mn toxicity treatment (5 ppm Mn^2+^, 3 weeks) at twelve days after transplanting. Mn toxicity caused moderate damage in rice in terms of biomass loss and symptom formation despite extremely high shoot Mn concentrations ranging from 2.4 to 17.4 mg g^-1^. The *tropical japonica* subpopulation was more sensitive to Mn toxicity than other subpopulations. Leaf damage symptoms were significantly correlated with Mn uptake into shoots. Association mapping was conducted for seven traits using 416741 single nucleotide polymorphism (SNP) markers using a mixed linear model, and detected six significant associations for the traits shoot manganese concentration and relative shoot length. Candidate regions contained genes coding for a heavy metal transporter, peroxidase precursor and Mn^2+^ ion binding proteins. The significant marker SNP-2.22465867 caused an amino acid change in a gene (LOC_Os02g37170) with unknown function. This study demonstrated significant natural variation in rice for Mn toxicity tolerance and the possibility of using GWAS to unravel genetic factors responsible for such complex traits.

## Background

Manganese (Mn) is an essential plant micro-nutrient participating in several metabolic pathways, including photosynthesis [1], and as a cofactor of several enzymes [2]. However, when accumulated in higher quantity, Mn can have phytotoxic effects [3]. High concentrations of Mn in the cell apoplast have been linked to the accumulation of phenoxy radicals and oxidized Mn^3+^, which are strong oxidizers of macromolecules such as lipids and proteins [4]. Therefore, Mn toxicity results in cell death and produces necrotic spots on leaves. Typical symptoms of Mn toxicity in rice are dark brown spots on crinkled lower leaves, chlorosis of young leaves and reduced growth [5].

Around 90% of rice is grown in lowland or flooded conditions [6], which is frequently subjected to nutrient disorders (toxicities or deficiencies). Among the most prominent nutrient disorders in flooded soil are iron (Fe) [7] and Mn toxicities [3]. When soils are submerged, oxygen is depleted by microbial respiration. Therefore, specific groups of microbes use transition metals such as Mn^4+^ and Fe^3+^ as a terminal electron acceptor to complete their energy metabolism. Microbial reduction of Mn^4+^ results in increased supply of the plant available form of reduced manganese (Mn^2+^) in the soil solution [7]. The amount of freely available Mn also increases with soil acidity [8].

The critical tissue Mn concentration threshold and toxicity tolerance varies among plant species [3,8]. Rice is considered as a rather Mn tolerant crop species [9–13]. Plants can achieve Mn toxicity tolerance either by exclusion or tissue tolerance [3]. Exclusion is supported by enhanced root oxidizing power or exudation of low molecular weight organic acids, which complex with freely available Mn [10]. Plants can also tolerate excess Mn in tissues by detoxification of excess Mn through sub-cellular partitioning. For example, Arabidopsis CAX2 (a putative vacuolar Ca^2+^/H^+^ antiport) plays an important role in the transport of Ca^2+^, Cd^2+^ and Mn^2+^ into the vacuoles [14,15]. Likewise, proteins of the cation diffusion facilitator family play an important role in metal tolerance in several organisms [16,17]. OsMTP8.1 in rice might be involved in Mn homeostasis by sequestering excess Mn into vacuoles of rice shoots [18]. A member of the yellow- stripe like protein family (OsYSL6) helps in the detoxification of excess Mn in the cell apoplast through the transport of Mn-nicotianamine complexes into the sub-cellular compartments [18]. Another mechanism of tissue tolerance to excess Mn is the scavenging of reactive oxygen species (ROS) through antioxidants, as suggested by high turnover of ascorbate in Mn tolerant cowpea and bean cultivars [19–21].

Only few studies have addressed genetic factors associated with Mn tolerance in crops. A single study reporting quantitative trait loci (QTL) for Mn toxicity tolerance in rice using a bi-parental mapping population has been published till date, in which eight QTL were identified [22]. However, bi-parental populations do not cover the enormous genetic diversity of Asian rice (*Oryza sativa* L.). Also, the resolution of bi-parental QTL mapping is low due to limited number of recombination [23]. Therefore, further studies exploring the genetic potential of adaption to high Mn concentrations in rice are required. Genome-wide association study (GWAS) is an alternative mapping strategy, which utilizes genetically diverse populations of unrelated individuals. Rice being a self-pollinating species, genotyping data generated for a mapping population can be reused to study the genetic architecture of different traits. Accordingly, a 44 k SNPs map for 413 rice genotypes was used to study aluminium toxicity tolerance [24], ozone tolerance [25], internal phosphorus use efficiency [26], iron toxicity tolerance [27] and boron toxicity tolerance [28]. A high density array (HDRA) of SNPs with 700 k polymorphic sites for 1536 rice genotypes was developed and verified by mapping loci associated with grain size [29]. Subsequently, HDRA data have been used to map genes associated with salinity tolerance [30] and cold tolerance [31].

Here we report a GWAS with the aim of identifying novel loci involved in Mn toxicity tolerance in rice and lining them to specific tolerance mechanisms. A mapping panel of 288 rice genotypes representing both *indica* and *japonica* sub-species were used for phenotypic screening in hydroponics under optimal and toxic Mn concentration. We used the 700 k SNPs genotyping data described previousely [29] to execute association mapping and identified candidate genes based on location, annotation, and functional polymorphisms.

## Materials and methods

### Plant materials

Seeds of a rice diversity panel were obtained from the International Rice Research Institute (IRRI, Los Baños, Philippines). In the phenotypic screening experiment 288 genotypes were used, but only 271 genotypes for which genotype data could be assigned were used in the subsequent mapping. The panel represented five sub-populations of Asian rice, i.e. 72 *indica*, 50 *aus*, 59 *tropical japonica*, 56 *temperate japonica* and 12 *aromatic* lines. Thirty nine lines were classed as admixture showing less than 80% ancestry from any single sub-group, i.e. *indica* or *japonica* [32].

### Phenotypic screening

A screening experiment was carried out in a greenhouse of the University of Bonn, Germany. Plants were grown in hydroponic culture for 33 days. An optimum rice growing environment was maintained in the greenhouse under natural light supplemented with artificial lighting for 12 hours a day to ensure a minimum light intensity of 19.5 kilo lux, 29/22°C day/night temperature, and 49/65 percent day/night relative humidity. Twelve-day-old rice seedlings were first transplanted into 60 liter tanks filled with half strength modified Yoshida solution [33]. Half strength solutions were replaced with full-strength solutions at 10 days after transplanting (DAT). The full strength composition of Yoshida solution was as follows: Nitrogen 40 ppm (NH_4_NO_3_), Phosphorus 10 ppm (NaH_2_PO_4_.2H_2_O), Potassium 40 ppm (K_2_SO_4_), Calcium 40 ppm (CaCl_2_), Magnesium 40 ppm (MgSO_4_.7H_2_O), Manganese 0.5 ppm (MnCl_2_.4H_2_O), Molybdenum 0.05 ppm [(NH_4_)_6_.Mo_7_O_24_.4H_2_O], Boron 0.2 ppm (H_3_BO_3_), Zinc 0.01 ppm (ZnSO_4_.7H_2_O), Copper 0.01 ppm (CuSO_4_.5H_2_O) and Iron 2 ppm (Fe-EDTA).

A preliminary experiment was conducted to establish an optimal Mn concentration that induces chronic Mn toxicity in rice. Seven genotypes were grown at five different Mn concentrations ranging from 0.5 ppm to 50 ppm. Manganese sulphate monohydrate (MnSO_4_.H_2_O) was used as a source for Mn. Plants were exposed to Mn treatments at 12 DAT and after three weeks of treatment plants were harvested and phenotyped as described below. Based on the result of preliminary experiment, a concentration of 5 ppm Mn was chosen for the main experiment, and excess Mn was supplied at 12 DAT to induce chronic Mn toxicity. The duration of the toxicity treatment was three weeks. The solution was replaced once a week to prevent nutrient depletion in the tanks, and the pH was adjusted to fall within a range of 5.2 to 5.5 twice a week. The experiment was set up as a completely randomized design. There were four independent replications per genotype per treatment, leading to a total of 32 hydroponic tanks, each accommodating up to 80 plants.

We scored leaf damage index (LDI) on the 20^th^ day after the start of the toxicity treatment on two tillers of each plant. First, the total numbers of fully expanded leaves were recorded, followed by counting of damaged leaves. Damaged leaf number was scored based on the extent of leaf area covered by symptoms. If two thirds or more area was covered with toxicity symptoms, they were scored as one leaf (1) otherwise half leaf (0.5). The ratio of the number of damaged leaves to the total leaves was recorded as LDI.

Plants were harvested on the 21st day after the beginning of the toxicity treatment. Tiller number, root length and shoot length were measured in each plant. Roots and shoots of individual plants were separated and dried at 60°C for three days for the determination of root and shoot dry weights.

We analysed shoot Mn concentration (SMC) for plants grown under toxic conditions. Dried shoots of all four biological replicates of each accession were pooled and finely ground. Sample extracts were prepared through pressure digestion using nitric acid (60%). An atomic absorption spectrophotometer was used to determine the concentration of Mn in the solution using a wavelength of 279.5 nm.

### Association mapping

Two alternative approaches were used to establish marker-trait associations and markers identified in both approaches were considered as significant. The 700 k SNP data used for mapping [29] provided approximately one SNP every 500 bp, covering all 12 chromosomes of rice. SNPs with minor allele frequency of less than 5% were removed [24, 27, 28], resulting in a total of 416741 SNPs. Maker information was retrieved for 271 rice genotypes, which were used for association mapping. To ensure normal distribution of phenotypic trait data, a squared-root transformation was applied for LDI, RSDW and log transformation was applied for RRDW and RTB.

First we used the statistical software package for R called rrBLUP [34]. This package uses a mixed linear model (MLM) with genotypic information as a random effect and population structure as fixed effects [35], thereby minimizing the risk of false association due to population stratification [36,37]. Five principal components and a kinship matrix were used to define the population structure in the model. rrBLUP returns a quantile-quantile plot and a Manhattan plot with a significant threshold set to a 5% FDR as calculated using the qvalue package [38].

In a second approach we adopted a MLM using the software Trait Analysis by Association, Evolution and Linkage (TASSEL) 5.0 of as described previously [39], adopting an arbitrary significance threshold of–log10(*p*)>5. The MLM incorporating PCA with five principal components and kinship data and was applied using the default settings (P3D for variance component analysis, compression level set to optimum level). Markers identified by both approaches were considered as true positives and analysed further for candidate gene selection.

### Candidate gene selection

With the 700 k SNP data used in this study, linkage disequilibrium (LD) decay is rapid within short distances between the SNP markers [29]. Therefore, declaration of candidate loci based on linkage disequilibrium blocks as adopted in previous studies using 44 k SNP data [24–28] might lead to false exclusion of candidate genes. Therefore, we applied a 200 kb window on either side of significant SNP to search putative candidate genes [40]. Gene models present in the candidate loci and their annotations were obtained from the MSU database (http://rice.plantbiology.msu.edu). Promising candidates were then classified based on the gene ontology, which was obtained from both the MSU database and UniProt database (http://www.uniprot.org/uniprot). The position and detailed allelic information of significant SNPs were retrieved from the allele finder window of Rice Diversity database (https://ricediversity.org/tools/). Tissue specific expression profile of candidate genes during different growth phase was obtained from the RiceXPro database (http://ricexpro.dna.affrc.go.jp) [41], and genes were excluded if no expression was reported in vegetative tissues. LD analysis was performed using Haploview 4.2 to observe the extent of LD in the regions harbouring significant markers. An LD block was created when upper confidence bounds of D’ value exceeded 0.98 and the lower bound exceeded 0.7 [42].

### Statistical analysis

We calculated relative phenotypic values (ratio of phenotypic value for plants grown under toxic Mn condition relative to control condition) for traits such as shoot length (relative shoot length, RSL), shoot dry weight (relative shoot dry weight, RSDW), root length (relative root length, RRL), root dry weight (relative root dry weight, RRDW), and subsequent statistical analyses were conducted using the program R [43]. Two-way ANOVA was conducted to observe the effects of genotype, treatment and genotype-treatment interaction on different traits. Also, Student’s *t*-test was conducted to compare the means of different traits for contrasting haplotypes. Multiple comparisons were performed using Tukey post hoc test. Pair-wise Pearson’s correlation coefficients between different phenotypes were estimated, and a linear regression model was used to display the relation between SMC and LDI.

## Results

### Genotypic variation for Mn toxicity tolerance in rice

**Fig 1:**
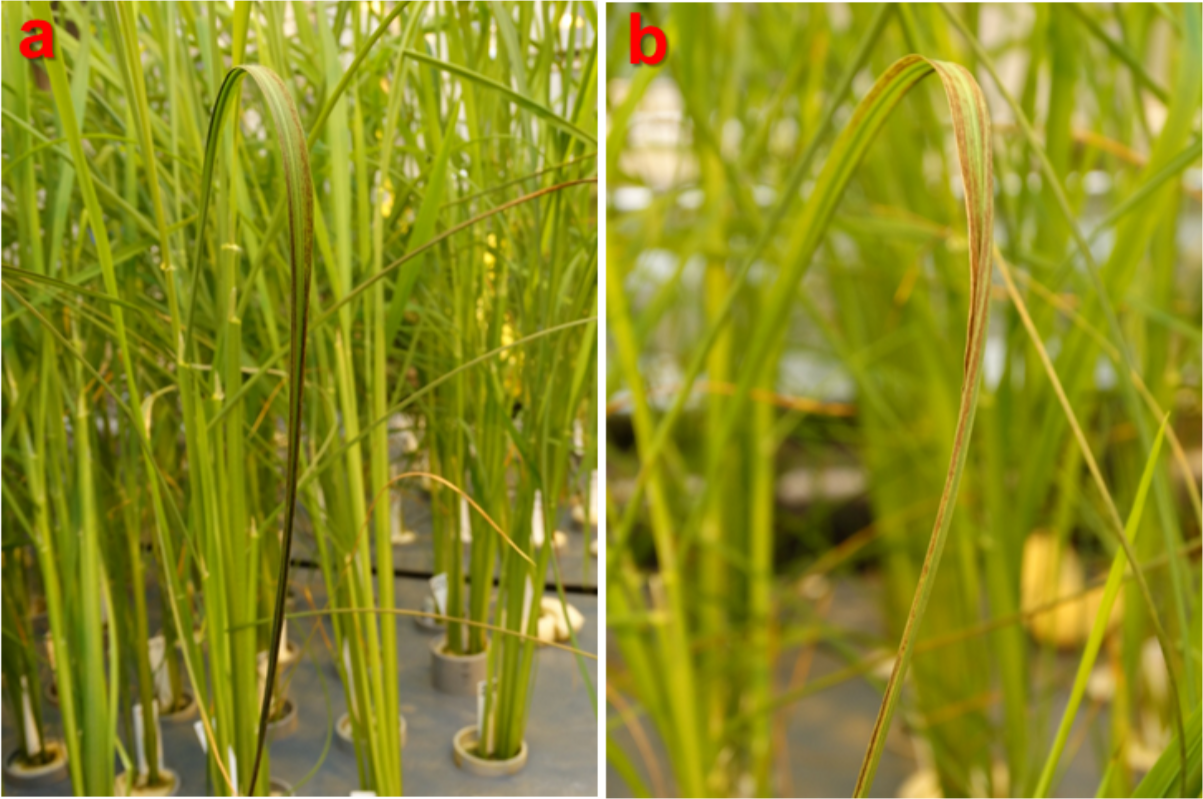
Leaf damage symptoms induced by Mn toxicity at 5 ppm for three weeks. Dark brown necrotic spots appear on curled fully expanded leaves. a Bombilla, *temperate japonica* from Spain. b Iguape Cateto, *tropical japonica* from Haiti.

**Fig 2:**
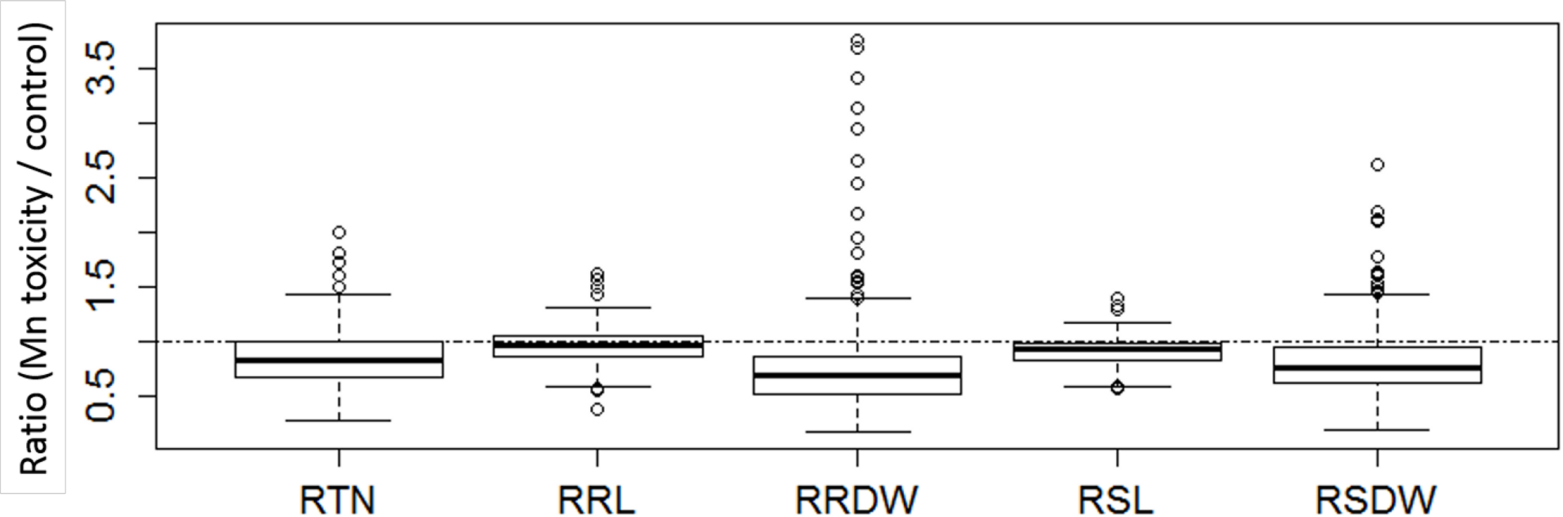
Box plot showing the distribution of relative phenotype values for relative tiller number (RTN), relative root length (RRL), relative root dry weight (RRDW), relative shoot length (RSL) and relative shoot dry weight (RSDW). Relative phenotypic value is the ratio of phenotypic value at toxic Mn condition/ control condition. The thick line in the middle of the box is the median of the distribution while the lower and upper boundaries represent first and third quartile, respectively. Lower and upper whiskers are calculated based on 1.5 times the inter-quartile range.

We evaluated seven traits including growth parameters, visual leaf damage symptoms (scored as LDI) and Mn concentration in shoots of plants grown under toxic Mn conditions. For plants grown under optimal Mn supply, all the phenotypes were scored except for shoot Mn concentration. After three weeks of chronic Mn stress, we observed significant symptom formation (Fig 1) and negative effects on biomass traits. When averaged over all genotypes, tiller number decreased by 18%, root length decreased by 9%, shoot length decreased by 4%, root dry weight decreased by 29% and shoot dry weight decreased by 21% (Table 1). Box plots for relative biomass traits (value in the stress treatment / value in the control) indicated large genotypic variation in response to Mn toxicity (Fig 2).

**Table 1:** Descriptive statistics and ANOVA results for different phenotypes.

Trait	Control (0.5 ppm Mn)			Treatment (5 ppm Mn)			ANOVA result		
	Min	Max	Mean	Min	Max	Mean	G	T	G*T
LDI (0–1)	0	0	0	0	1	0.368	***	NA	NA
Tiller number	1	7	1.88	1	8	1.54	***	***	***
Shoot length (cm)	29	118	84.25	20	120.5	76.6	***	***	***
Root length (cm)	9	39.5	23.23	4.5	42	22.09	***	***	***
Shoot dry weight (g)	0.12	2.94	0.87	0.04	2.85	0.68	***	***	***
Root dry weight (g)	0.02	0.58	0.16	0.01	0.41	0.11	***	***	***
SMC (mg g^-1^)	ND	ND	ND	2.41	17.43	6.8	***	NA	NA

Significance levels are indicated at *p*<0.001/***. Treatment and genotype*treatment interaction effects were not analyzed for leaf damage index (LDI) as there were no leaf damage symptoms on plants grown under control condition. Shoot Mn concentration (SMC) for plants grown under control condition was not determined. G genotype, Max maximum, Min minimum, NA not applicable, ND not determined, T treatment

**Fig 3:**
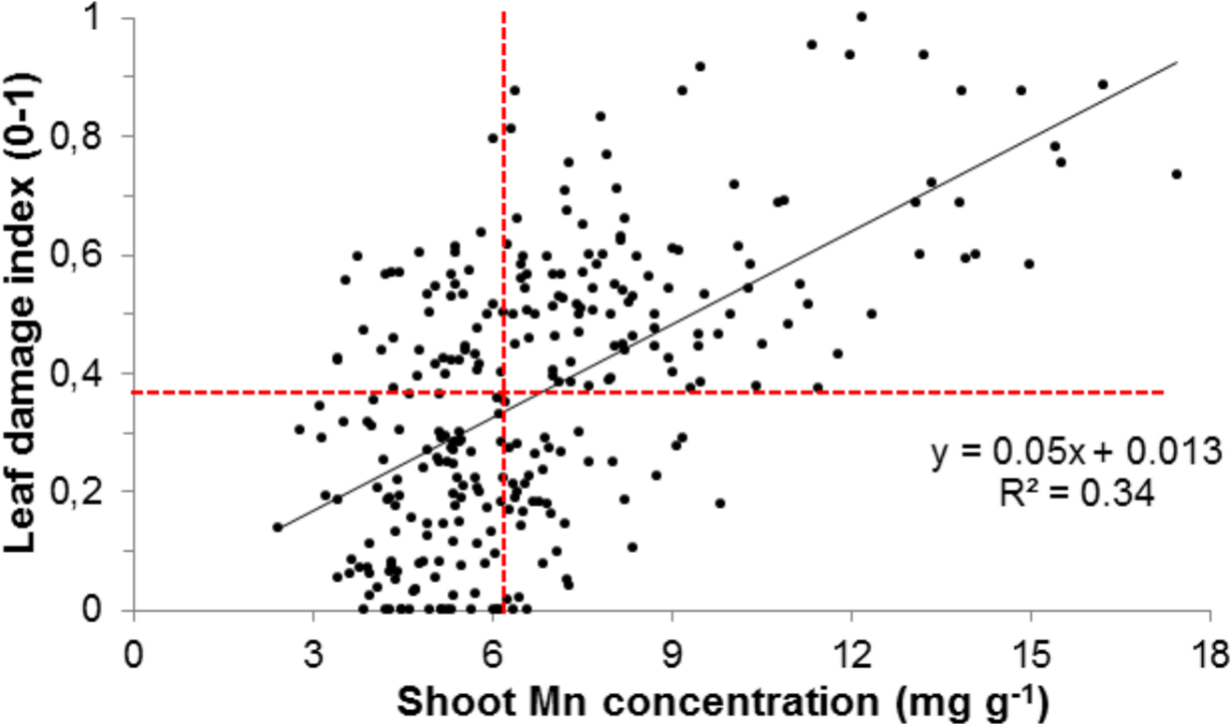
Linear regression of shoot Mn concentration and leaf damage index. Estimated gradient for the explanatory variable (shoot Mn concentration) was unequal to zero (*p*<0.001). Horizontal and vertical dotted red lines represent the median values for leaf damage index and shoot Mn concentration, respectively.

Moreover, significant correlations between several phenotypic traits were observed. SMC was negatively correlated with most traits except visible leaf damage symptoms, in which a positive correlation was seen. However, no significant correlations were observed between RTN and SMC (Table 2). Linear regression suggested that 34 percent of the variation observed in leaf damage was explained by Mn accumulation (Fig 3). The median values for SMC and LDI were 6.25 mg g^-1^ and 0.38, respectively, and were incorporated into the linear regression model. We observed that among the genotypes that had LDI below the median (i.e. the more tolerant half of the population) the majority had SMC below the median. This indicated that Mn exclusion was the primary tolerance mechanism in this mapping population, although a substantial number of genotypes had ‘tissue tolerance’, showing low leaf damage despite high SMC (Fig 3). Further, we evaluated the effect of excess Mn in different sub-populations. We observed that visual leaf damage was the lowest for the *indica* sub-population and the highest for *tropical japonica* (Fig A in S1 File). As expected, shoot Mn concentration was also highest for *tropical japonica* (Fig B in S1 File). The differences in phenotypic response can be attributed to the strong population stratification between rice sub-populations [24,32], and points out the importance of considering population structure in association mapping.

**Table 2:** Pair-wise correlation coefficients between phenotypes.

	LDI	RTN	RRL	RSL	RRDW	RSDW	SMC
LDI		0.011	<0.001	<0.001	<0.001	<0.001	<0.001
RTN	-0.15		<0.001	0.054	<0.001	<0.001	0.566
RRL	-0.33	0.26		<0.001	<0.001	<0.001	<0.001
RSL	-0.59	0.11	0.49		<0.001	<0.001	<0.001
RRDW	-0.41	0.53	0.46	0.59		<0.001	<0.001
RSDW	-0.53	0.57	0.5	0.69	0.87		<0.001
SMC	0.58	-0.03	-0.32	-0.65	-0.32	-0.42	

The lower triangle shows the Pearson correlation coefficient for each pair-wise comparison. The upper triangle shows the probability of correlation coefficient for a pair of phenotypes being equal to zero. LDI leaf damage index, RRDW relative root dry weight, RRL relative root length, RSDW relative shoot dry weight, RSL relative shoot length, RTN relative tiller number, SMC shoot Mn concentration

### Association mapping

**Fig 4:**
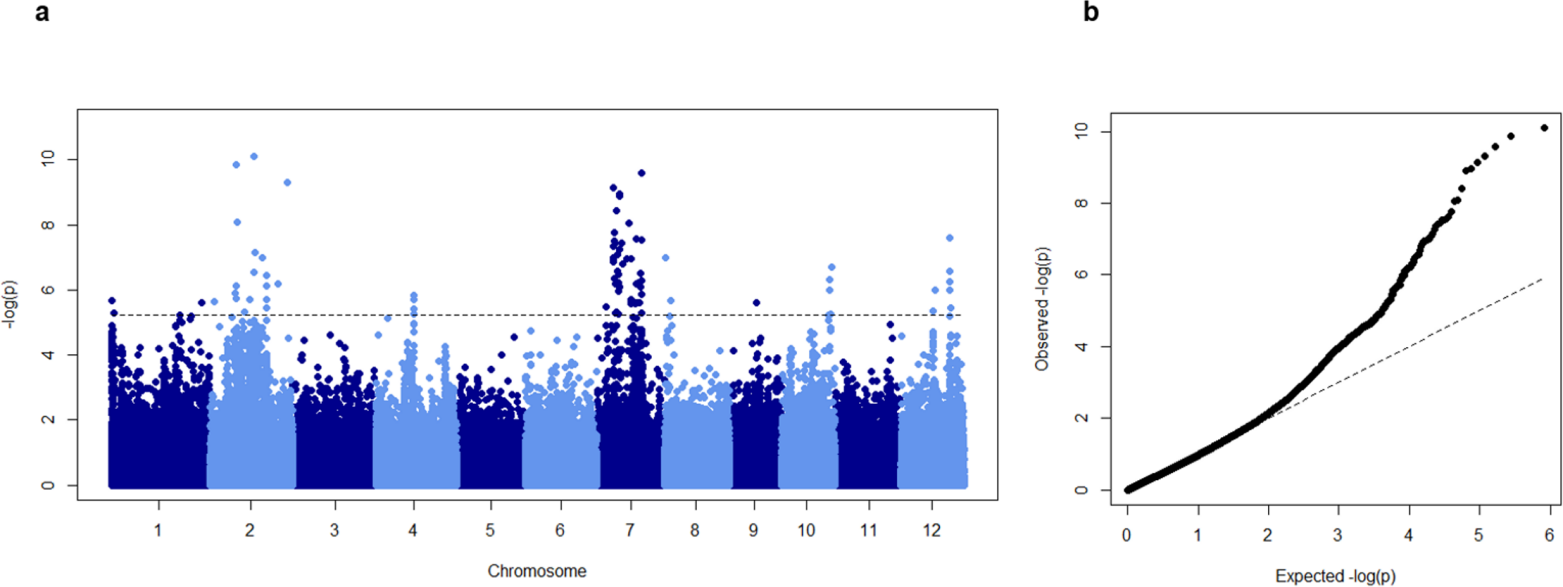
Association mapping result for shoot Mn concentration (SMC). A Manhattan plots from association mapping using mixed linear model. X-axis shows the SNPs along the 12 chromosomes of rice and Y-axis shows -log_10_ (*p*) value of association for each SNP. b Quantile-quantile plot of expected and observed -log_10_ (*p*).

**Fig 5:**
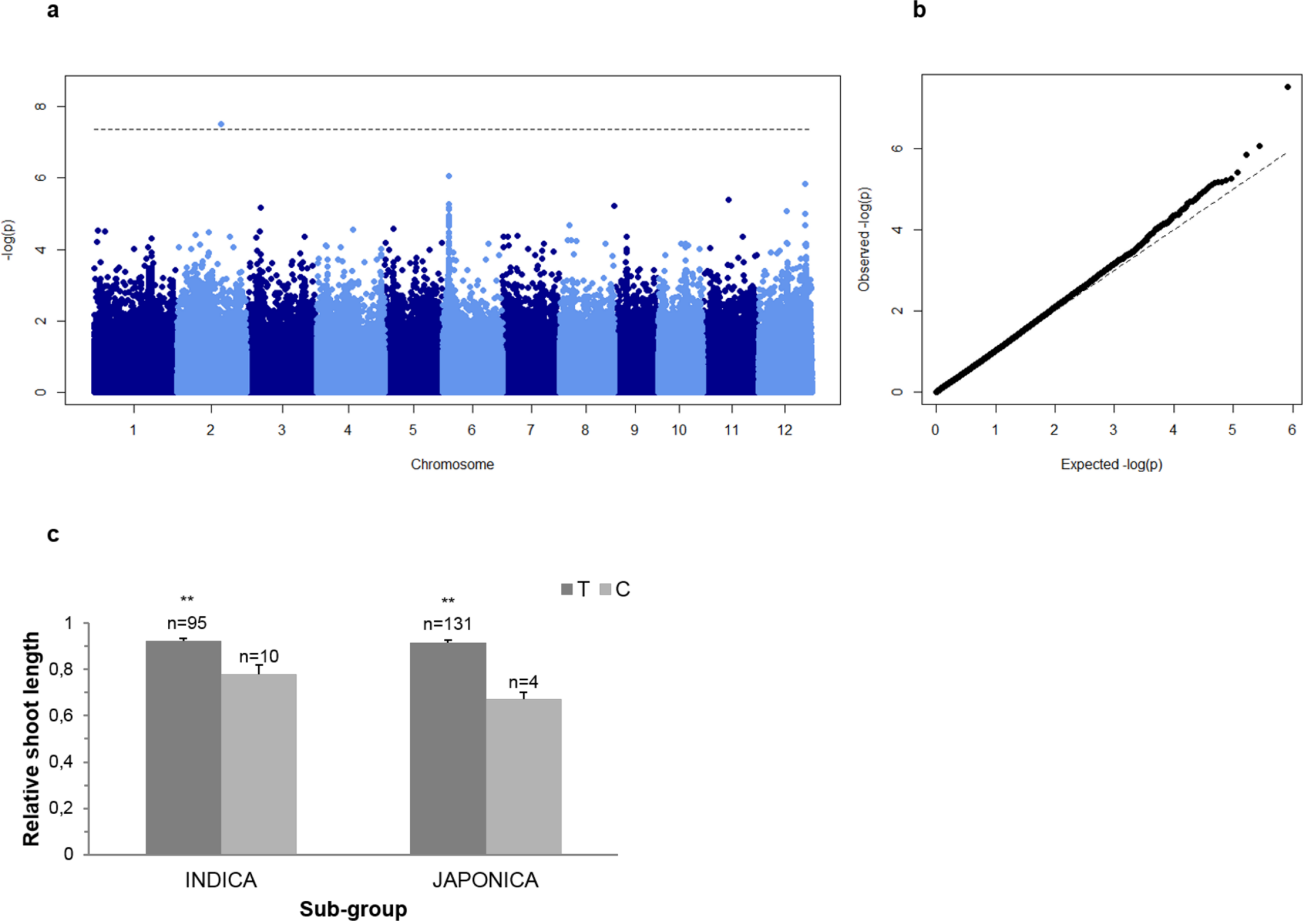
Association mapping result for relative shoot length (RSL). A Manhattan plots from association mapping using mixed linear model. X-axis shows the SNPs along the 12 chromosomes of rice and Y-axis shows -log_10_ (*p*) value of association for each SNP. b Quantile-quantile plot of expected and observed -log_10_ (*p*). c Haplotypes for a significant SNP (SNP-2.22465867) located in the coding sequence of LOC_Os02g37170. Haplotypes are separated by *indica* and *japonica* sub-group. The number above the bars indicates lines per haplotype. Significance levels are indicated with **/ *p*<0.01. Error bars indicate standard error.

The phenotypic values and frequency distribution of traits used for association mapping is presented in Table A in S2 File and Fig C in S1 File. First, we performed a principal component analysis [44] in order to detect the presence of population structure. The first two principal components (PCs) explained around 21% of the total genetic variation (Fig D in S1 File), and demonstrated a clear subpopulation structure reflecting the different subpopulations of rice. Therefore, five principal components and a kinship matrix were in the model to explain the population stratification. Association mapping was performed using rrBLUP [34,35] to identify loci linked to the evaluated traits. Eighty SNP passed the 5% FDR threshold. To further validate these results significant SNPs were compare to those that passed a significance threshold of [-log_10_ (*p*)>5] in TASSEL 5.0 analysis. The latter results are presented as supplementary data (Figs E and F in S1 File). This approach led to significant associations for two traits: RSL and SMC (Figs 4 and 5). A list of genes contained in candidate loci is presented as supplementary material (Table B in S2 File).

#### SMC

Association mapping for SMC detected 5 significant SNPs on chromosomes 2 and 12 (Fig 4A). The gene LOC_Os12g35040 annotated as 2,3-bisphosphoglycerate-independent phosphoglycerate mutase was located 16.5 kb downstream of SNP-12.21274465. This protein has a Mn^2+^ binding ligand and catalyzes the inter-conversion of 2-phosphoglycerate and 3-phosphoglycerate [45]. Also, genes involved in signal transduction (LOC_Os12g35320) and cellular redox homeostasis (LOC_Os12g35330 and LOC_Os12g35340) were present within a 150 kb window of SNP-12.21274465 (Table B in S2 File and Fig G in S1 File). A cytochrome P450 gene (LOC_Os12g25660) involved in iron ion binding was located in candidate loci harbouring SNP-12.14960076 (Table B in S2 File and Fig H in S1 File). An ATPase, AAA family protein (LOC_Os02g19450) involved in abiotic stress signalling was located around 30 kb apart from SNP-2.11394332 on chromosome 2 (Table B in S2 File and Fig I in S1 File). Similarly, two Mn^2+^ ion binding proteins (LOC_Os02g29000 and LOC_Os02g29020) annotated for abiotic stress response were present in 66 kb window with significant SNP-2.17092835 (Table B in S2 File and Fig J in S1 File). Moreover, a glutamate receptor (LOC_Os02g54640), a MYB transcription factor (LOC_Os02g54520) and a serine threonine kinase (LOC_Os02g54590) were located within candidate loci harbouring SNP-2.33444628 (Table B in S2 File and Fig K in S1 File). Several markers with higher -log_10_ (*p*) value were detected on chromosome 7 because the significant SNPs were in strong LD (Fig L in S1 File). The region harboured plausible candidates such as the heavy metal transporters OsNRAMP5 (LOC_Os07g15370) and OsNRAMP1 (LOC_Os07g15460).

#### RSL

A significant SNP-2.22465867 on chromosome 2 was located in the coding sequence of LOC_Os02g37170, and resulted in Asp-Gly substitution (Haplotype analysis presented in Fig 5C). A copper transporter LOC_Os02g37160 and several putative heavy metal transporter proteins were present in the region harbouring SNP-2.22465867 (LOC_Os02g37190, LOC_Os02g37280, LOC_Os02g37290, LOC_Os02g37300, LOC_Os02g37320 and LOC_Os02g37330) (Table B in S2 File).

## Discussion

In our preliminary experiment, we found that Mn concentrations from 2 to 10 ppm in nutrient solutions were sufficient to induce chronic Mn stress in rice. A wide range of Mn concentrations between 200 μM / 11 ppm [10] to 2 mM / 110 ppm [5] was previously used in hydroponics to induce Mn toxicity in rice. We opted for a moderate Mn concentration (5 ppm) for our GWAS to induce a chronic Mn stress level, in which substantial genotypic differences for all evaluated traits and reduced growth in response to Mn toxicity were observed (Table 1). Necrosis of leaves and decreased growth was previously reported in rice under excess Mn conditions in hydroponics experiments [5,13,18]. In addition, we observed significant negative correlation between LDI and all biomass traits (Table 2), similar to previous experiments, in which a diverse rice population was screened for boron toxicity tolerance in rice [28]. Therefore, cell death symptoms produced by these toxicities can serve as reliable indicators of response to stress. Oxidative damage during Mn toxicity was previously shown to be linked to elevated production of highly reactive phenoxy radicals and accumulation of Mn^3+^ in the apoplast [8,19,46]. Therefore, tolerance might also be explained by the metal concentration in the tissue. In our study, shoot Mn concentration varied between 2.4 mg g^-1^ in the cultivar Lomello, *temperate japonica* and 17.4 mg g^-1^ in the cultivar NPE844, *tropical japonica*. Mn hyper-accumulators such as *Virotia neurophylla*, *Chengiopanax sciadophylloides* and *Maytenus spp*. can tolerate foliar Mn concentration as high as 25 mg g^-1^ [47]. Some herbaceous plants such as *Alternanthera philoxeroides* and *Phytolacca acinosa* can also tolerate more than 7 mg g^-1^ of foliar Mn [48]. However, cultivated crop plants are mostly sensitive to excess Mn. Barley showed leaf necrosis and reduced growth at foliar Mn concentrations between 170 and 280 μg g^-1^ [49]. Similarly, soybean [50], cowpea and beans [20] are very sensitive to Mn toxicity. That some of the rice genotypes in this screening study showed very little damage despite excessively high shoot Mn concentration confirms previous studies [9, 11–13] stating that rice can be considered as a rather Mn tolerant species. It has been previously reported that rice can tolerate Mn levels up to 5 mg g^-1^ without showing any damage symptoms [11,18]. In our study, we found that some tolerant rice genotypes tolerated even higher concentrations of Mn in shoots (SMC>6.2 mg g^-1^, median value) (Fig 3). Notably, we observed significant positive correlation between shoot metal concentration and leaf symptoms, which was similar to a previous study on iron toxicity [27] but different from a study on boron toxicity in rice [28]. This may indicate that exclusion mechanisms play a more eminent role with toxicities of transition metals such as Mn and Fe.

In this study we employed a HDRA composed of 700 k SNPs [29] for a sub-set of population that had previously been genotyped using 44 k SNP [32]. The diversity panel showed deep sub-population structure based on 700 k SNP genotyping data. The five sub-populations of rice formed clear clusters similar to observations by McCouch et al. (2015) [29] and Zhao et al. (2011) [32], highlighting the importance of using a mixed model in the mapping order to account for population structure. The use of a HDRA is expected to improve the detection power and mapping resolution of GWAS [29,30]. For example, no significant associations were detected for salinity tolerance in rice with 44 k SNP data, while the HDRA SNP data detected multiple significant SNPs [30]. In our study, two alternative models identified six consensus SNPs linked to two different traits. We calculated LD of regions containing significant markers and assigned LD blocks for co-segregation of markers [42]. LD decay was rapid with distance and the size of LD blocks harbouring significant SNP varied between 8.7 kb to 666 base pairs. Most of these small LD blocks were located in intergenic regions that contained no gene models (Table C in S2 File). Even though rice is a self-pollinating crop with potentially slower LD decay, the high density of markers will capture many historical recombination events which might contribute to LD decay [51]. Also, the HDRA possibly captures isolated mutation events in certain groups of the rice population contributing to LD decay. Because LD block analysis was not helpful in determining candidate regions in our study, we applied a 200 kb window on either side of significant SNP to look for putative candidate genes linked to the traits as previously suggested [40]. However, it needs to be considered that a fixed window approach might cause false inclusion or exclusion of candidate genes [25,52].

Among the candidate genes identified in this study, a SNP variant detected for RSL causing an amino acid substitution was located right in the coding sequence of LOC_Os02g37170 (Table 3). Although the function of this gene and its involvement in Mn metabolism is unknown, it should be considered that many genes that have been demonstrated to underlie agronomically important QTLs in rice, such as *Dro1* [53] or *Pup1* [54], were unknown or regulatory proteins. In the case of this candidate gene, the tolerant allele was the dominant one (Fig 5C) and may unintentionally have been favoured by breeders due to the selective pressure of high soluble Mn levels in paddy rice fields.

**Table 3:** Summary of significant single nucleotide polymorphisms (SNPs) markers for two traits: Shoot manganese concentration (SMC) and relative shoot length (RSL).

Trait	SNP	Chr	Position	Allele			Remark
				Major	Minor	Missing	
SMC	SNP-2.11394332.	2	11394337	A (210)	G (61)		Intergenic region
SMC	SNP-2.17092835.	2	17098706	G (154)	A (76)	41	Intergenic region
SMC	SNP-2.33444628.	2	33450498	A (139)	G (119)	13	Intergenic region
SMC	SNP-12.14960076.	12	14962735	T (195)	C (35)	41	Intergenic region
SMC	SNP-12.21274465.	12	21307919	G (250)	C (14)	6	Intergenic region
RSL	SNP-2.22465867.	2	22471737	T (226)	C (15)	31	Gene coding, non-synonymous, Asp-Gly, LOC_Os02g37170

SNPs which passed the 5% FDR adjustment (rrBLUP) and -log_10_ (*p*)>5 (TASSEL) are listed together with the corresponding trait, SNP marker ID (used in HDRA SNP genotype data), chromosome (Chr) number and position. Retrotransposon genes were not considered in the analysis. SMC shoot Mn concentration, RSL relative shoot length

Some of the candidate genes contained in candidate loci can be assigned to typical adaptive strategies employed by plants. The transport of Mn between plant organs plays an important role in Mn tolerance. *OsNRAMP5* (LOC_Os07g15370) is known to be involved in root to shoot translocation of Mn in rice [55,56] and was listed among the genes associated with SMC. Among the candidate genes associated with SMC was also *OsNRAMP1* (LOC_Os07g15460), an orthologue of *AtNRAMP1* which has been characterized as a high affinity Mn transporter regulating Mn uptake in Arabidopsis [57].

Shoot tolerance is generally referred to as the ability to tolerate high concentrations of elements in aboveground tissue through inclusion mechanisms. Mn shoot tolerance was illustrated in a bi-parental QTL mapping study, where the tolerant parent (IR1552, *indica*) accumulated more Mn in rice shoots than the sensitive one [22]. Tissue tolerance can be achieved through various mechanisms such as sub-cellular detoxification of Mn, and scavenging of ROS [3]. YSL6 proteins and cation diffusion facilitators (MTP) have been characterized to play key roles in Mn homeostasis in rice and Arabidopsis through sub-cellular transport of excess Mn [13,17,18,58,59]. Among the candidate genes identified in this study, transition metal ion transporters (LOC_Os02g37190, LOC_Os02g37280, LOC_Os02g37290, LOC_Os02g37300, LOC_Os02g37320 and LOC_Os02g37330) were located in a significant locus on chromosome 2 which might help in detoxification of excess Mn^2+^. These genes are highly expressed in both roots and shoots especially in early vegetative stages (http://ricexpro.dna.affrc.go.jp). Moreover, proteins with metal binding domains are known to confer heavy metal toxicity tolerance [60]. Three Mn^2+^ binding proteins (LOC_Os12g35040, LOC_Os02g29000, LOC_Os02g29020) were present in the candidate loci for SMC in this study. Likewise, antioxidant activity is important for the scavenging ROS, which can arise from reactions involving transition metals such as Mn [61–63]. The involvement of plants’ antioxidant systems with Mn tolerance was previously demonstrated in rye [64] and cowpea [19]. Two of the candidate genes for SMC detected in this study were annotated as OsGRX- glutaredoxin (LOC_Os12g35320 and LOC_Os12g35340) on chromosome 12. *OsGRX* genes were constitutively expressed in shoots in vegetative growth stages (http://ricexpro.dna.affrc.go.jp). GRX is involved in maintaining glutathione dependent peroxidase activity as part of the antioxidant system during oxidative stress in plants [65–67].

## Conclusions

This study provided novel insight into the natural variation in Mn toxicity tolerance in rice. It clearly demonstrated that most rice varieties tolerate quite high levels of Mn in their tissue, an ability that may have developed during the evolution and domestication of the species on highly reducing soils. A generally high level of tolerance is also reflected in the fact that for several highly significant SNP markers detected in this study, the major alleles were more tolerant than the minor alleles. Nevertheless, substantial variability was observed in all stress related phenotypes, which offers opportunities for further optimizing Mn tolerance in rice. The study also demonstrated that both inclusion and exclusion tolerance mechanisms are important under Mn toxicity. A number of interesting candidate genes related to Mn exclusion, transport and partitioning as well as cellular redox homeostasis, and stress response were detected through association mapping. This study advances our understanding of Mn tolerance in rice and other crops, and will contribute to the breeding of crops adapted to Mn toxic conditions.

## Supporting information

S1 File Supplementary figures. Fig A–Fig L.
(PPTX)

S2 File Supplementary tables. Table A–Table C.
(XLSX)
