# Supplementary material for: Genome-wide association study to identify candidate loci and genes for Mn toxicity tolerance in rice
Source: PLoS One. 2018 Feb 9;13(2):e0192116. doi: 10.1371/journal.pone.0192116 (PMC5806864; doi:10.1371/journal.pone.0192116)
Supplement: S1 File — Fig A–Fig L. (PPTX) [file pone.0192116.s001.pptx]

## Slide 1
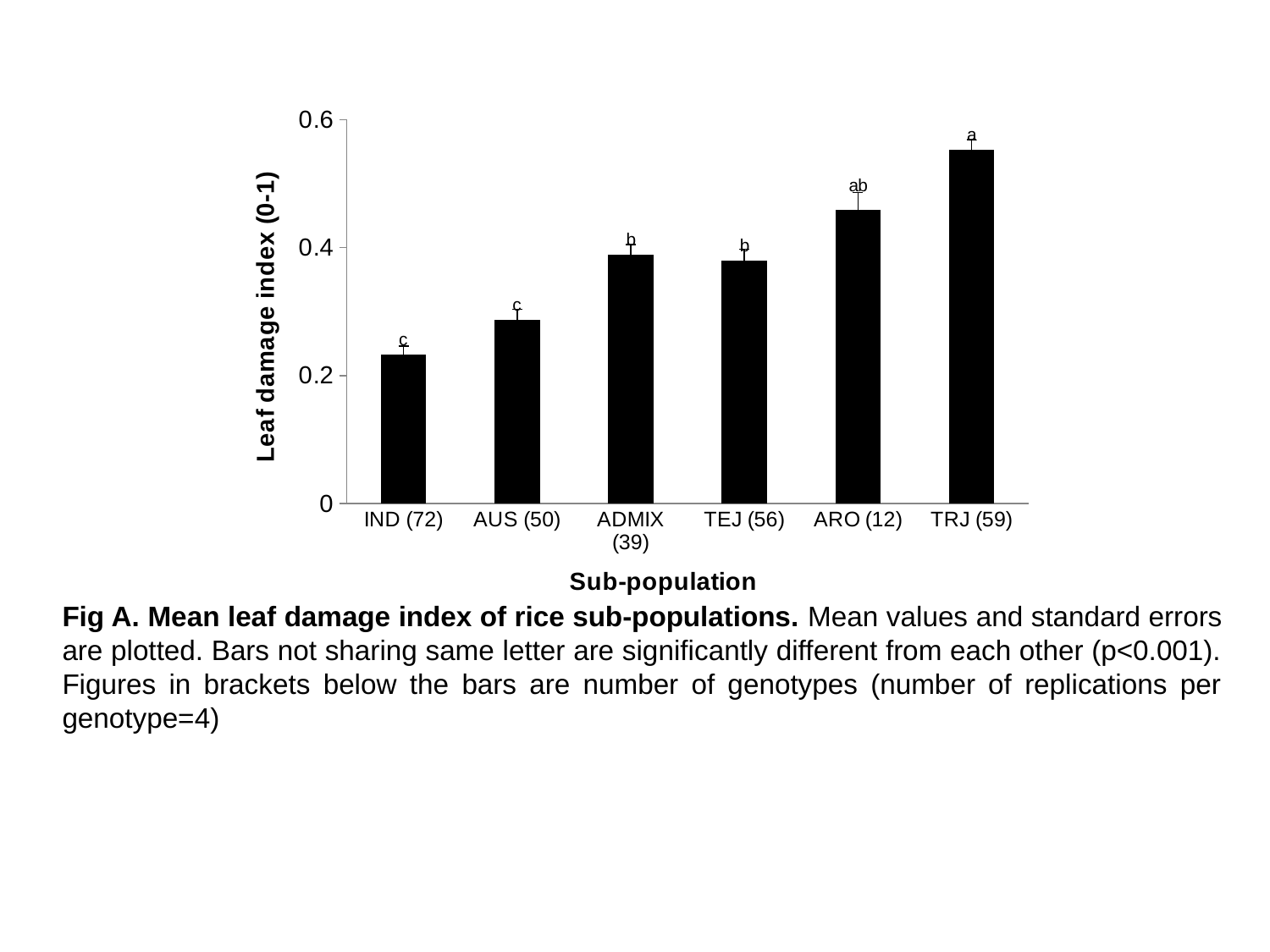

### Chart
| Category | ldi |
|---|---|
| IND (72) | 0.23249221318101002 |
| AUS (50) | 0.2872341037779633 |
| ADMIX (39) | 0.38910027750027776 |
| TEJ (56) | 0.3798624589893249 |
| ARO (12) | 0.45947916666666677 |
| TRJ (59) | 0.5525982782050748 |Fig A. Mean leaf damage index of rice sub-populations. Mean values and standard errors are plotted. Bars not sharing same letter are significantly different from each other (p<0.001). Figures in brackets below the bars are number of genotypes (number of replications per genotype=4)

## Slide 2
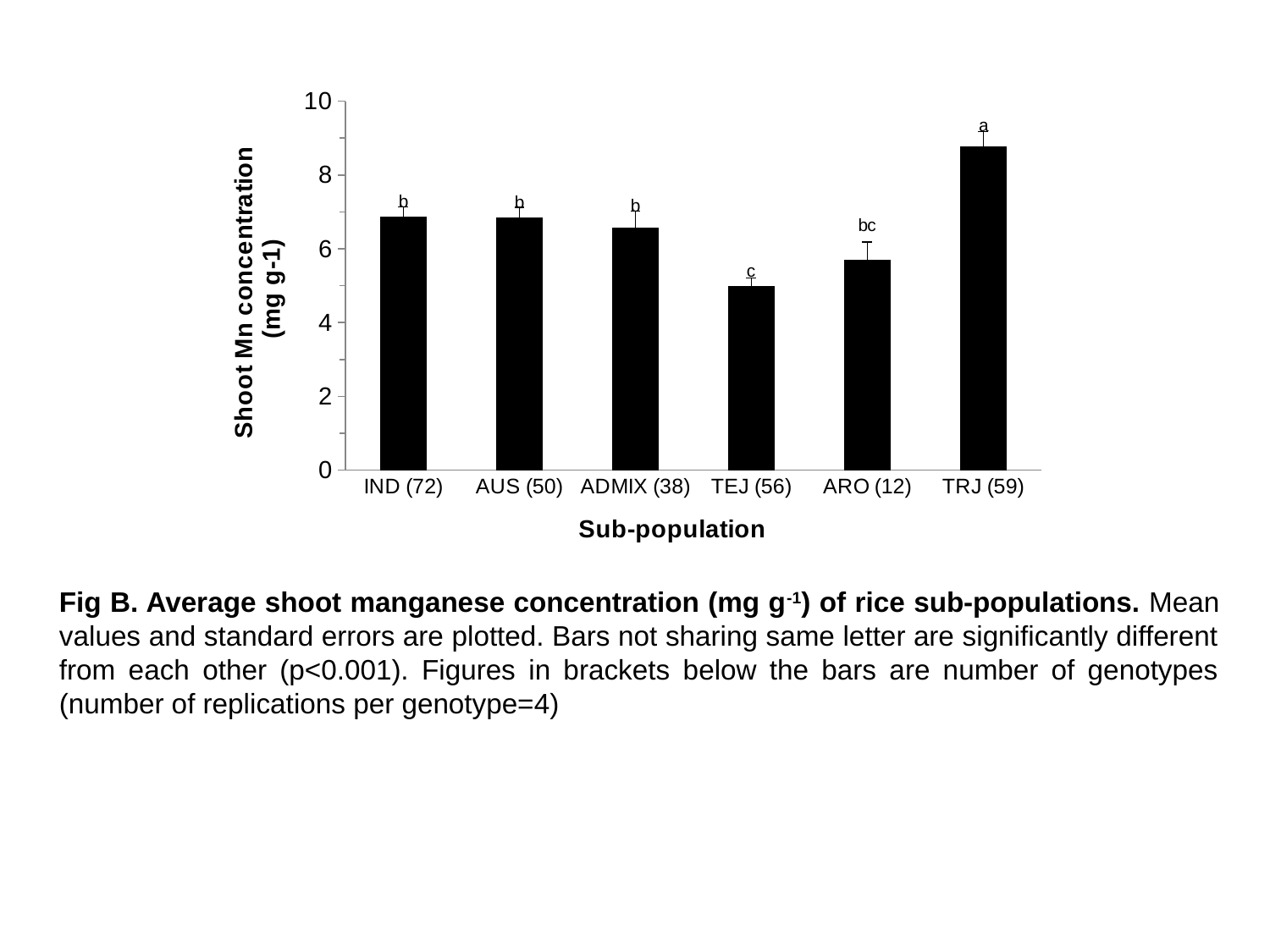

### Chart
| Category | Shoot Mn content (mg Mn/ g DM) |
|---|---|
| IND (72) | 6.8720080784919375 |
| AUS (50) | 6.858670666820293 |
| ADMIX (38) | 6.569164781241399 |
| TEJ (56) | 5.001917175216968 |
| ARO (12) | 5.6980311985463254 |
| TRJ (59) | 8.764375456370644 |Fig B. Average shoot manganese concentration (mg g-1) of rice sub-populations. Mean values and standard errors are plotted. Bars not sharing same letter are significantly different from each other (p<0.001). Figures in brackets below the bars are number of genotypes (number of replications per genotype=4)

## Slide 3
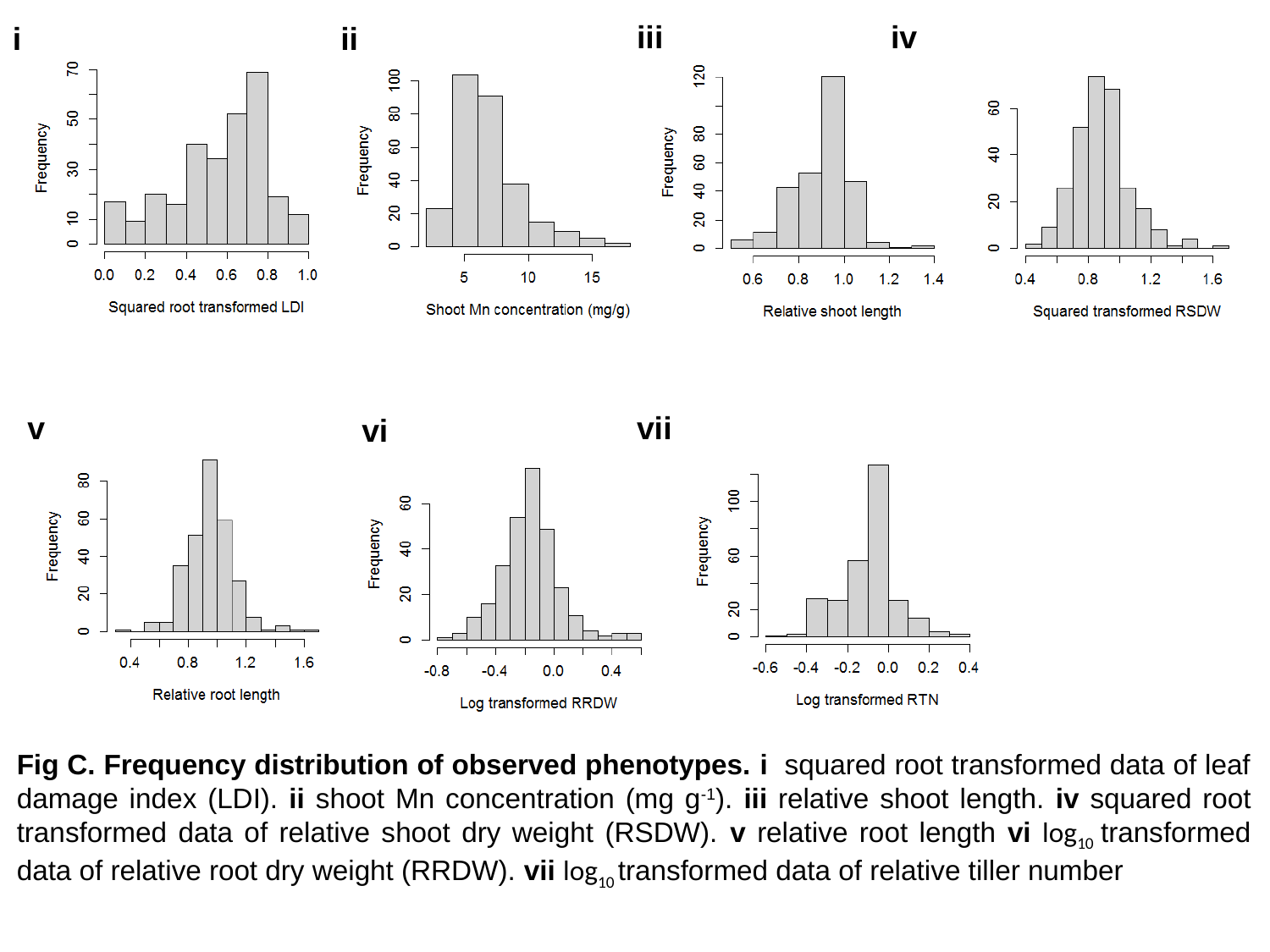

iii
iv
i
ii
v
vii
vi
Fig C. Frequency distribution of observed phenotypes. i squared root transformed data of leaf damage index (LDI). ii shoot Mn concentration (mg g-1). iii relative shoot length. iv squared root transformed data of relative shoot dry weight (RSDW). v relative root length vi log10 transformed data of relative root dry weight (RRDW). vii log10 transformed data of relative tiller number

## Slide 4
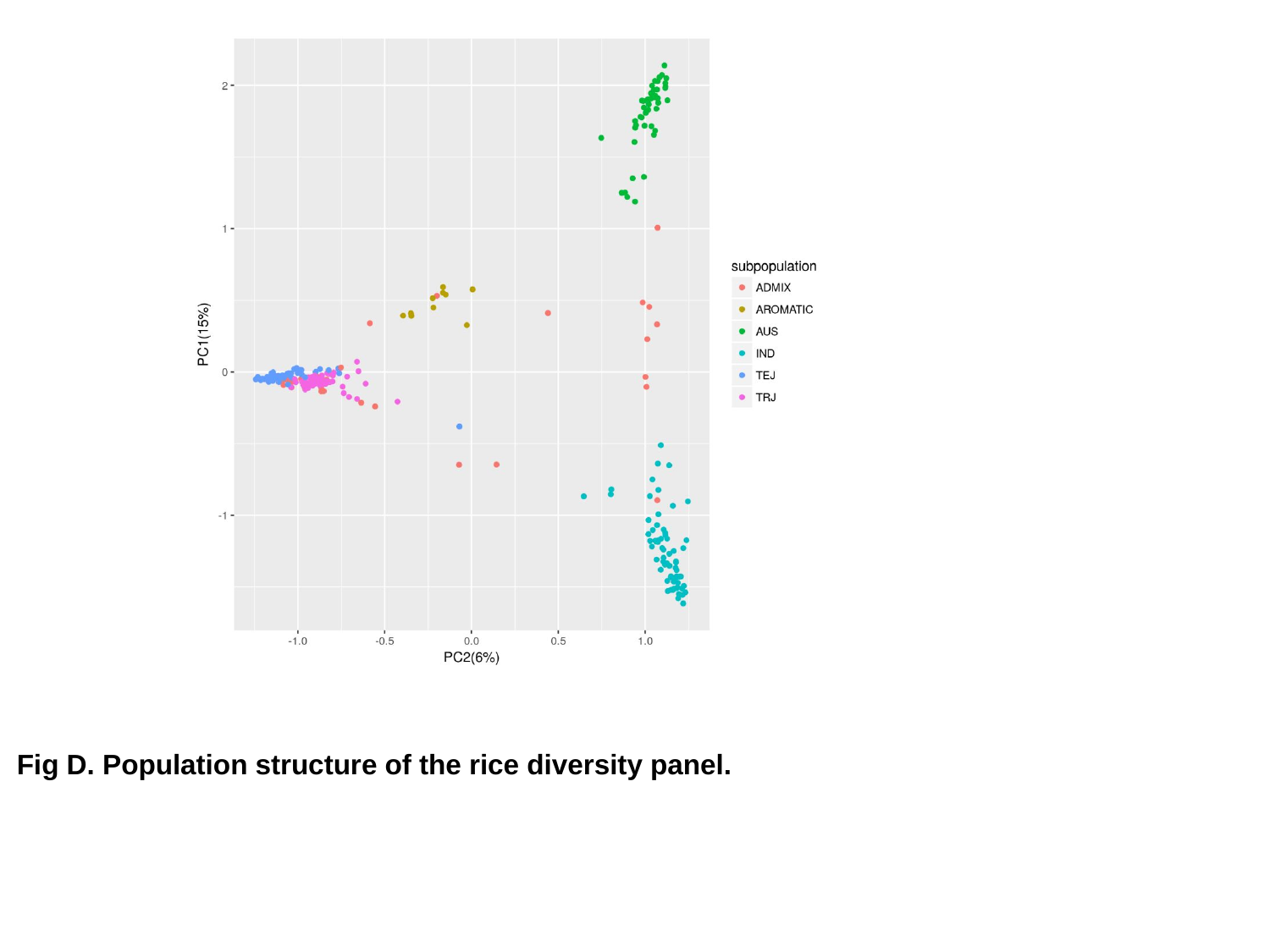

Fig D. Population structure of the rice diversity panel.

## Slide 5
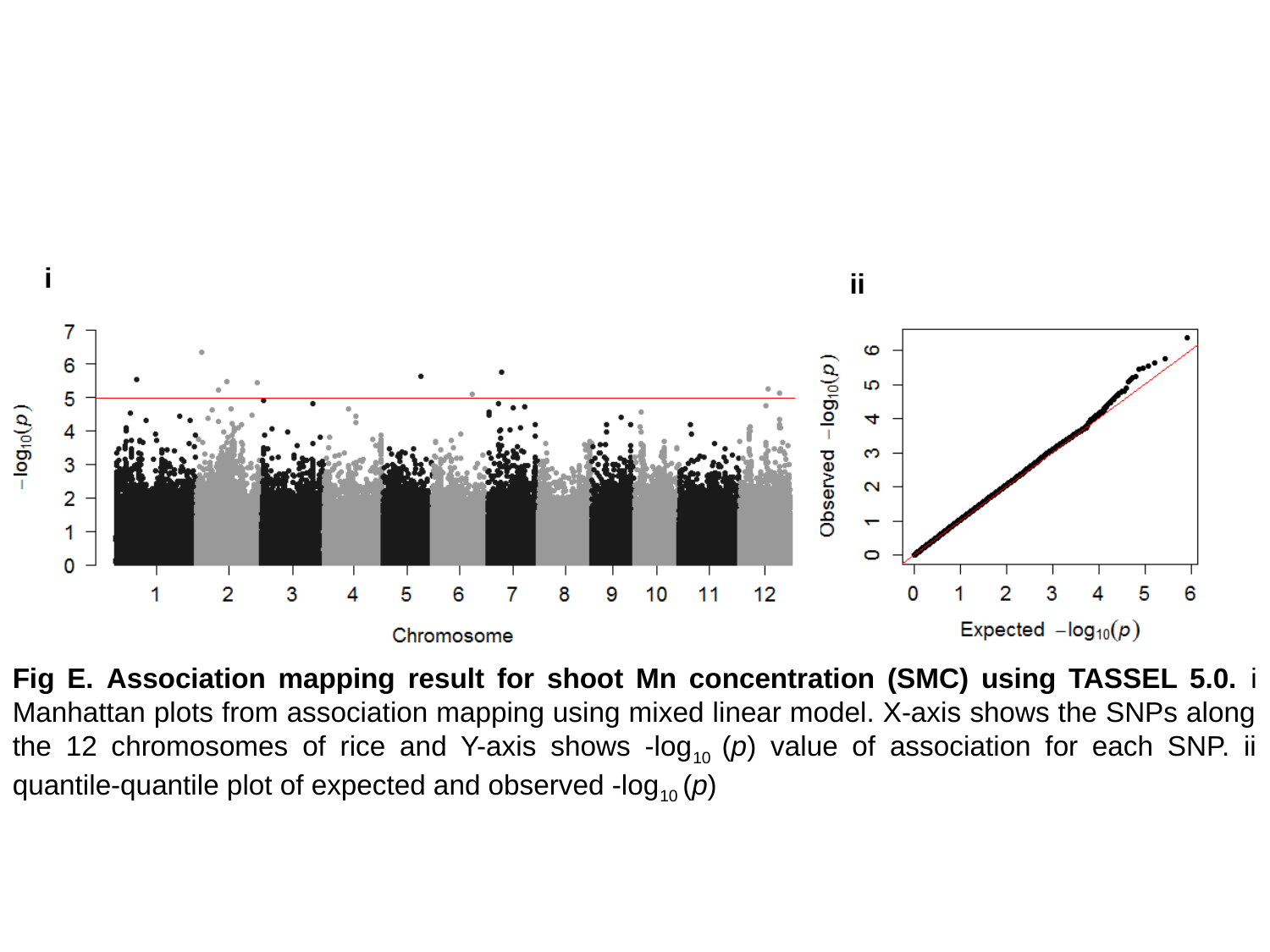

i
ii
Fig E. Association mapping result for shoot Mn concentration (SMC) using TASSEL 5.0. i Manhattan plots from association mapping using mixed linear model. X-axis shows the SNPs along the 12 chromosomes of rice and Y-axis shows -log10 (p) value of association for each SNP. ii quantile-quantile plot of expected and observed -log10 (p)

## Slide 6
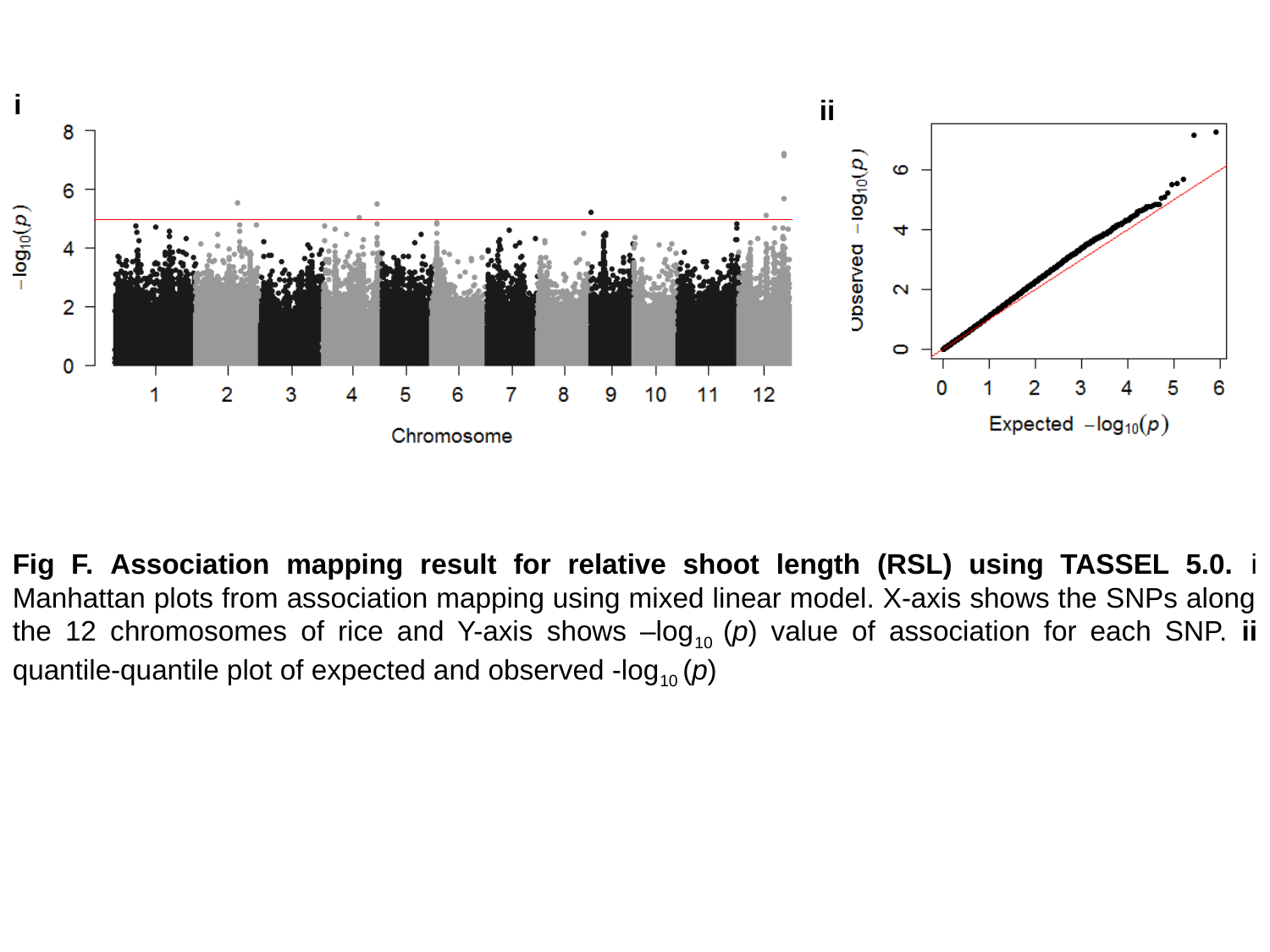

i
ii
Fig F. Association mapping result for relative shoot length (RSL) using TASSEL 5.0. i Manhattan plots from association mapping using mixed linear model. X-axis shows the SNPs along the 12 chromosomes of rice and Y-axis shows –log10 (p) value of association for each SNP. ii quantile-quantile plot of expected and observed -log10 (p)

## Slide 7
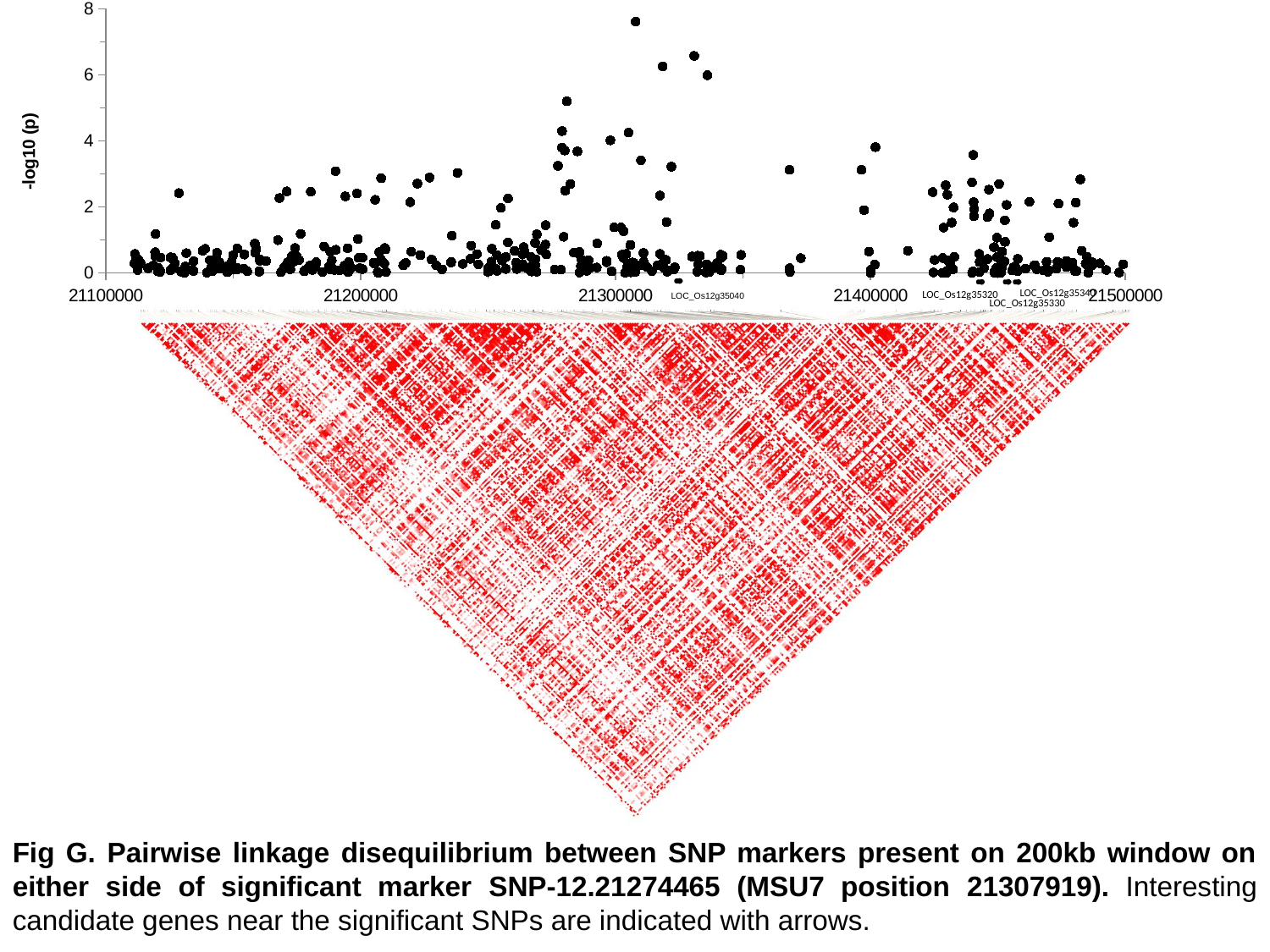

### Chart
| Category | logp |
|---|---|
LOC_Os12g35340
LOC_Os12g35320
LOC_Os12g35040
LOC_Os12g35330
Fig G. Pairwise linkage disequilibrium between SNP markers present on 200kb window on either side of significant marker SNP-12.21274465 (MSU7 position 21307919). Interesting candidate genes near the significant SNPs are indicated with arrows.

## Slide 8
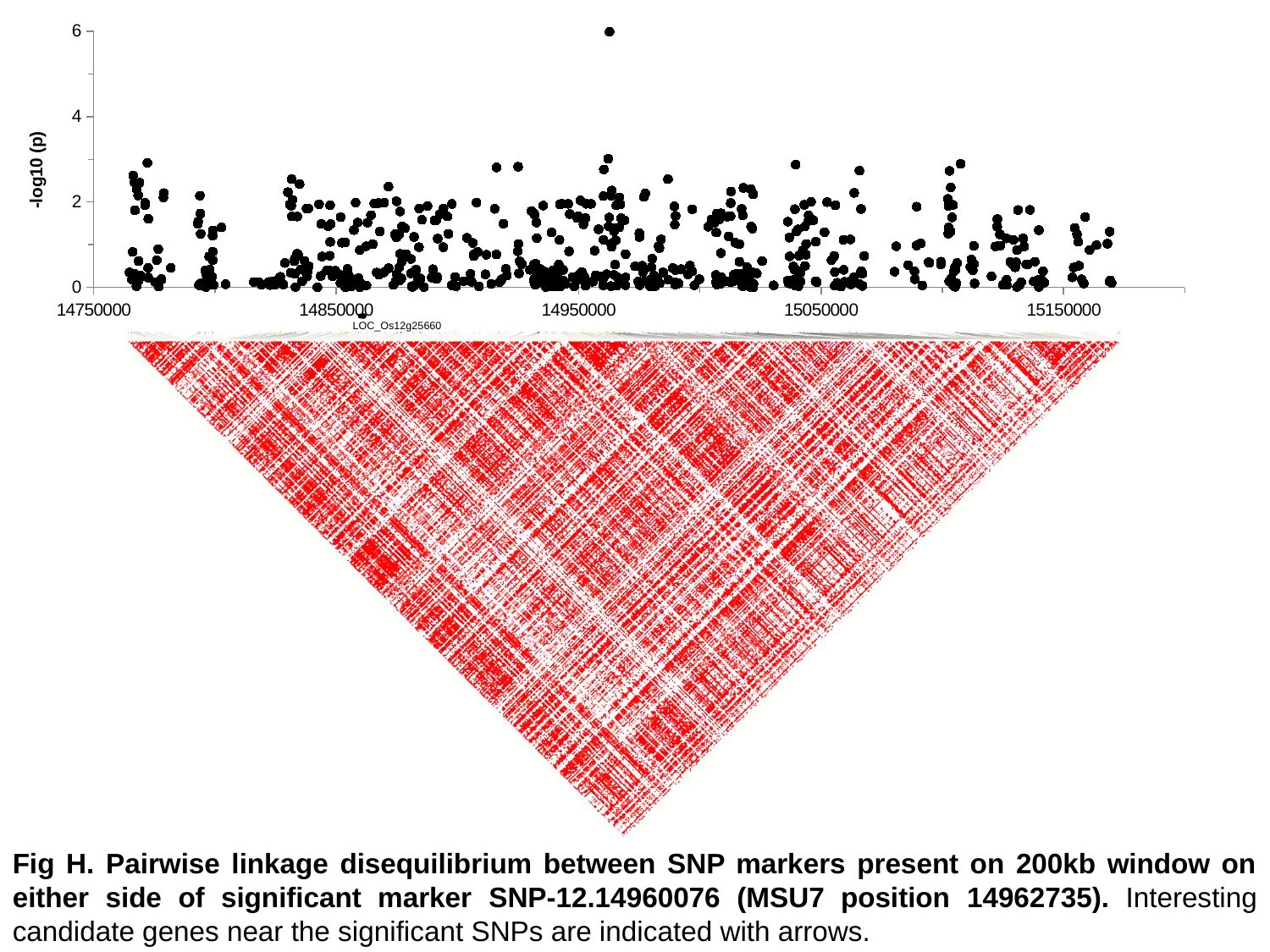

### Chart
| Category | logp |
|---|---|
LOC_Os12g25660
Fig H. Pairwise linkage disequilibrium between SNP markers present on 200kb window on either side of significant marker SNP-12.14960076 (MSU7 position 14962735). Interesting candidate genes near the significant SNPs are indicated with arrows.

## Slide 9
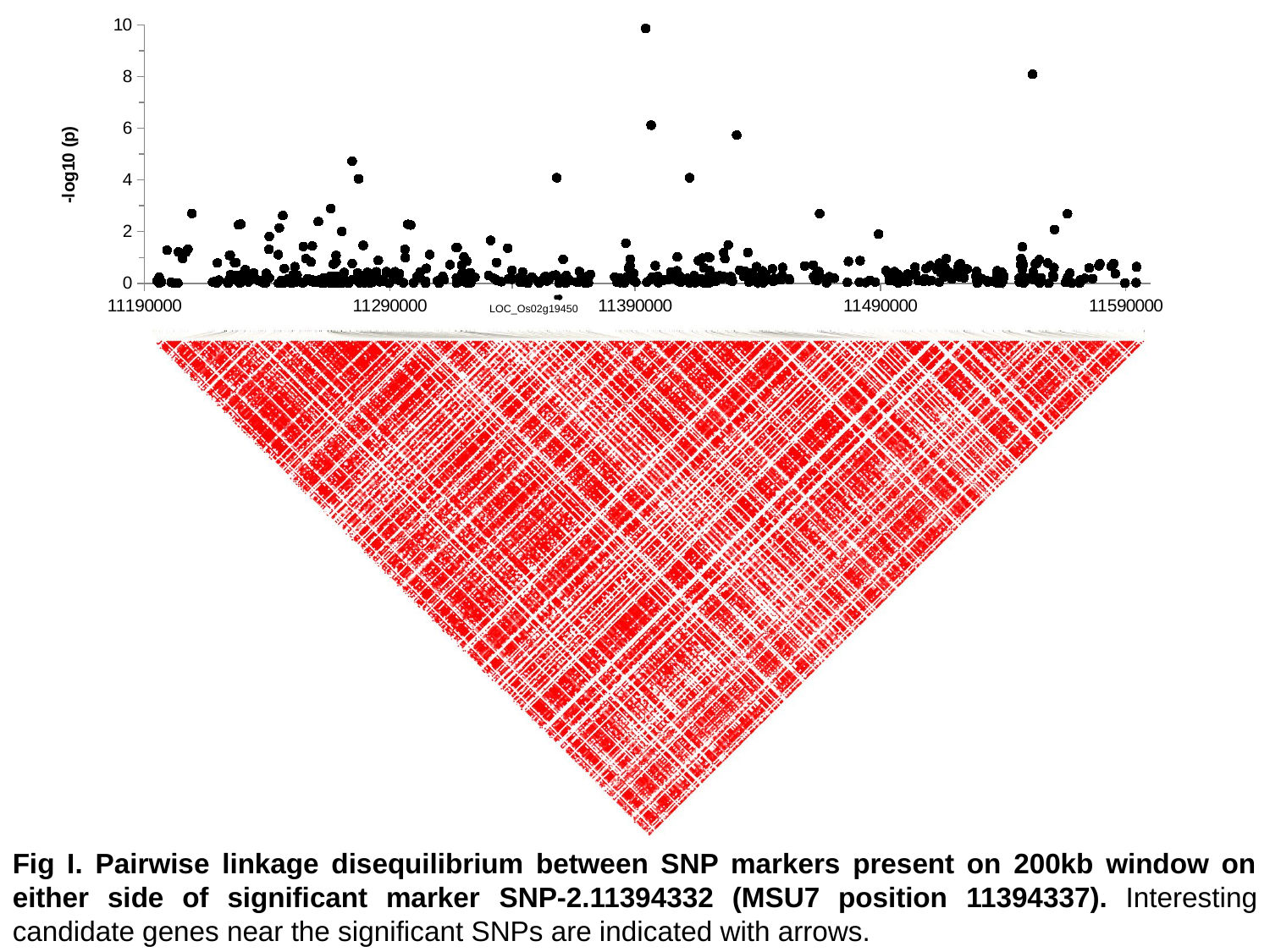

### Chart
| Category | logp |
|---|---|
LOC_Os02g19450
Fig I. Pairwise linkage disequilibrium between SNP markers present on 200kb window on either side of significant marker SNP-2.11394332 (MSU7 position 11394337). Interesting candidate genes near the significant SNPs are indicated with arrows.

## Slide 10
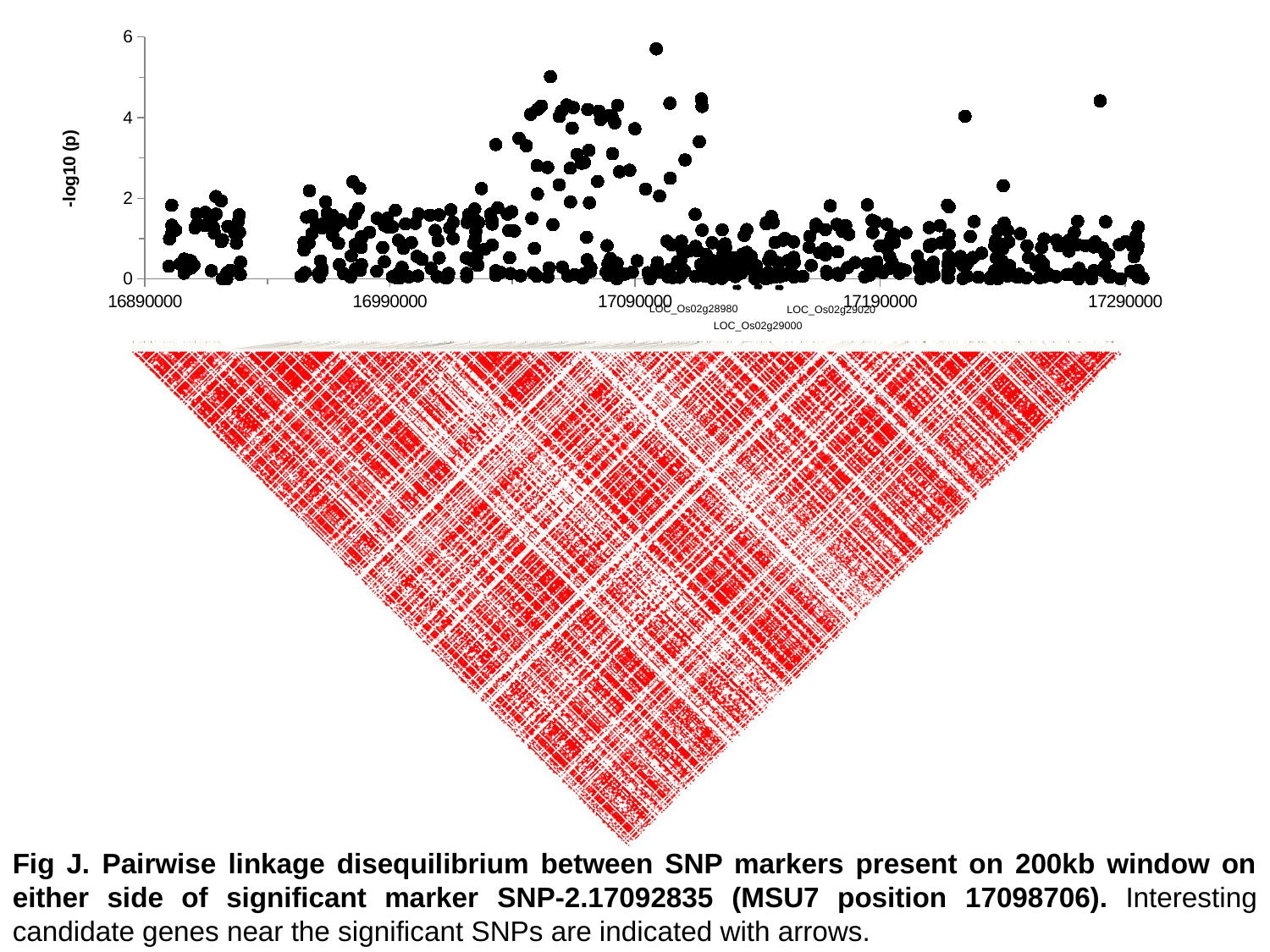

### Chart
| Category | logp |
|---|---|
LOC_Os02g28980
LOC_Os02g29020
LOC_Os02g29000
Fig J. Pairwise linkage disequilibrium between SNP markers present on 200kb window on either side of significant marker SNP-2.17092835 (MSU7 position 17098706). Interesting candidate genes near the significant SNPs are indicated with arrows.

## Slide 11
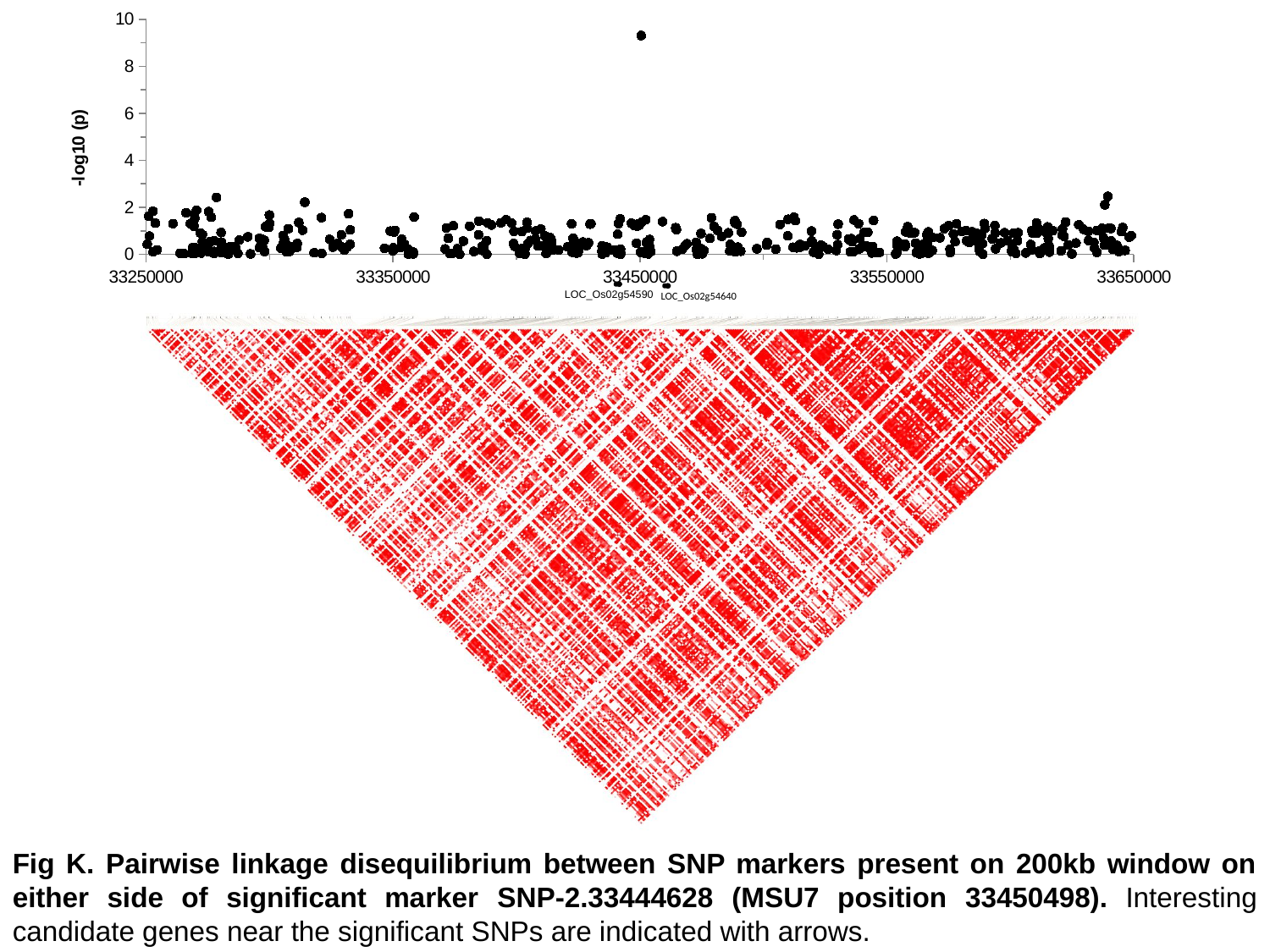

### Chart
| Category | logp |
|---|---|LOC_Os02g54590
LOC_Os02g54640
Fig K. Pairwise linkage disequilibrium between SNP markers present on 200kb window on either side of significant marker SNP-2.33444628 (MSU7 position 33450498). Interesting candidate genes near the significant SNPs are indicated with arrows.

## Slide 12
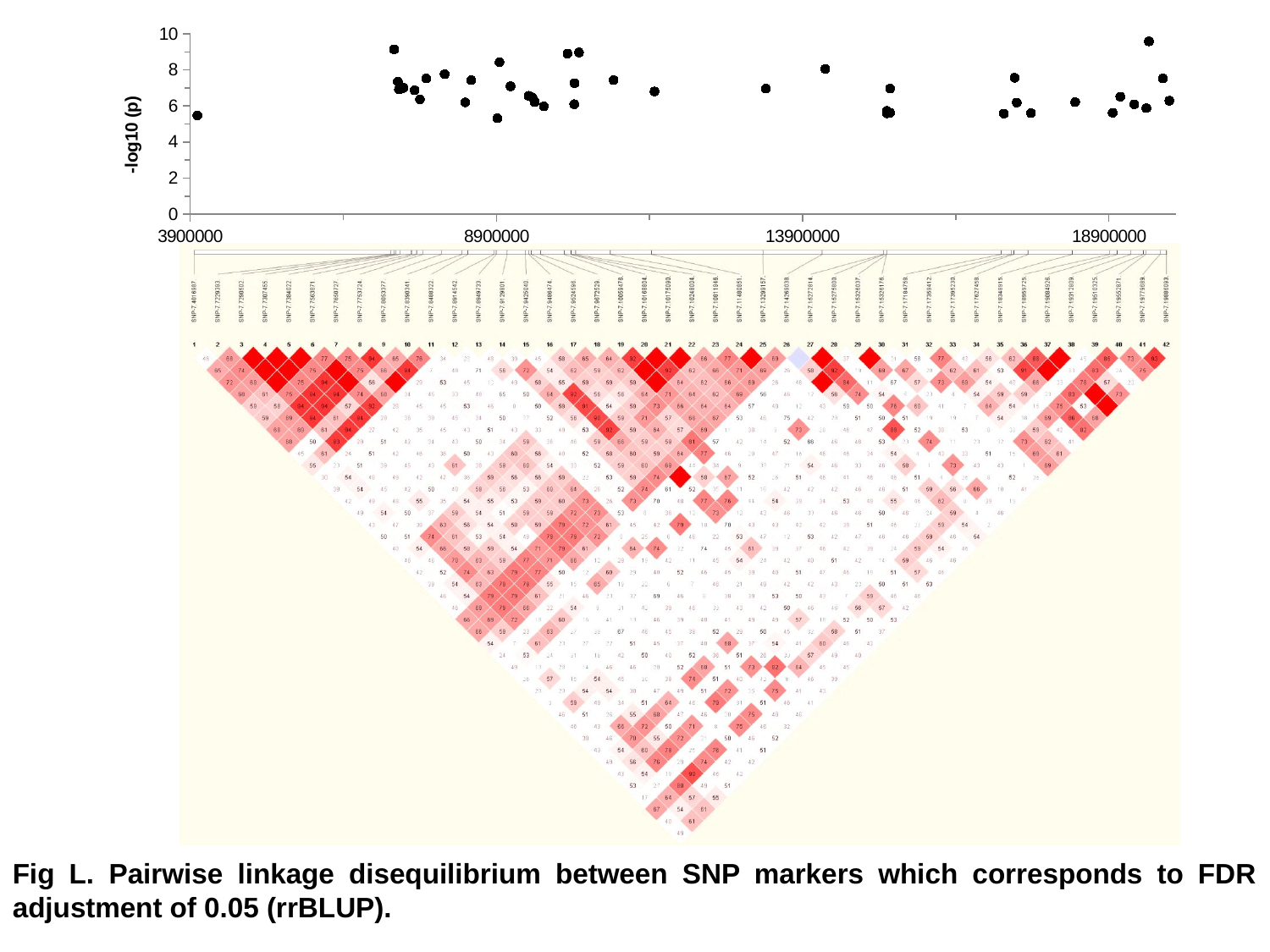

### Chart
| Category | |
|---|---|
Fig L. Pairwise linkage disequilibrium between SNP markers which corresponds to FDR adjustment of 0.05 (rrBLUP).
